# Towards deployable CRISPR-based nucleic acid detection

**DOI:** 10.1088/2516-1091/ae6e0b

**Published:** 2026-06-02

**Authors:** Andrew Guo, Alexandra G Bell, Cameron Myhrvold

**Affiliations:** 1 Department of Molecular Biology, Princeton University, Princeton, NJ 08544, United States of America; 2 Omenn-Darling Bioengineering Institute, Princeton University, Princeton, NJ 08544, United States of America; 3 Department of Chemical and Biological Engineering, Princeton University, Princeton, NJ 08544, United States of America; 4 Department of Chemistry, Princeton University, Princeton, NJ 08544, United States of America

**Keywords:** CRISPR, nucleic acid detection, infectious disease, sample processing, multiplexing, isothermal amplification, deployable diagnostics

## Abstract

Deployable diagnostics are necessary for the control and treatment of infectious diseases, with significant unmet needs revealed during the COVID-19 pandemic. Nucleic acid diagnostics remain among the most sensitive and specific forms of detection, yet their reliance on laboratory equipment and trained personnel limits their deployment in resource limited settings. CRISPR-based diagnostics are uniquely positioned to enable rapid, affordable, and highly accurate nucleic acid testing at both the point-of-care and the point-of-need. In this review, we discuss advances toward deployable CRISPR-based diagnostics. We begin by examining innovations in sample processing methods, emphasizing strategies that reduce equipment requirements and enhance compatibility across diverse sample types and pathogens. We then explore developments in one-pot isothermal and amplification-free approaches, comparing the benefits and tradeoffs associated with each, as well as multiplexing strategies for simultaneous detection of multiple pathogens. Finally, we consider additional factors that impact assay deployability, including reagent lyophilization to minimize cold chain dependence and readout technologies that enable detection in resource-limited settings. We conclude by outlining remaining challenges and opportunities for future progress.

## 1. Introduction

Fast and accurate diagnostics are critical for infectious disease monitoring, supporting both clinical decision making and robust public health responses [1]. For many infectious diseases, nucleic acid diagnostics are considered the gold standard, valued for their accuracy of detection even in the earliest stages of disease [2–4]. The COVID-19 pandemic underscored the importance of nucleic acid diagnostics for outbreak control, yet their utility extends beyond human health [5]. Nucleic acid diagnostics are additionally used for food safety monitoring, agricultural pathogen management, and environmental biosensing [6–8].

Techniques based on either polymerase chain reaction (PCR) or sequencing have been widely adopted for sensitive detection of nucleic acids. However, their reliance on specialized equipment, such as thermocyclers or sequencing machines, and trained personnel prevents their integration into resource-limited environments, such as in point-of-care (POC) settings, where testing is done at the site of patient care, or in even more constrained point-of-need (PON) settings, where testing is done without any clinical support or infrastructure such as at home [9, 10]. Isothermal (i.e. single temperature) amplification methods help bypass restrictive equipment requirements while also streamlining diagnostic workflows, making them potentially suitable for POC and PON applications. However, their accuracy is often affected by the amplification of non-specific targets, and their ability to discriminate single nucleotide differences is limited [11, 12]. To this end, there is an unmet need for POC and PON nucleic acid diagnostics that achieve the accuracy of PCR-based methods and the simplicity and cost-effectiveness of isothermal amplification methods.

**Figure 1.**
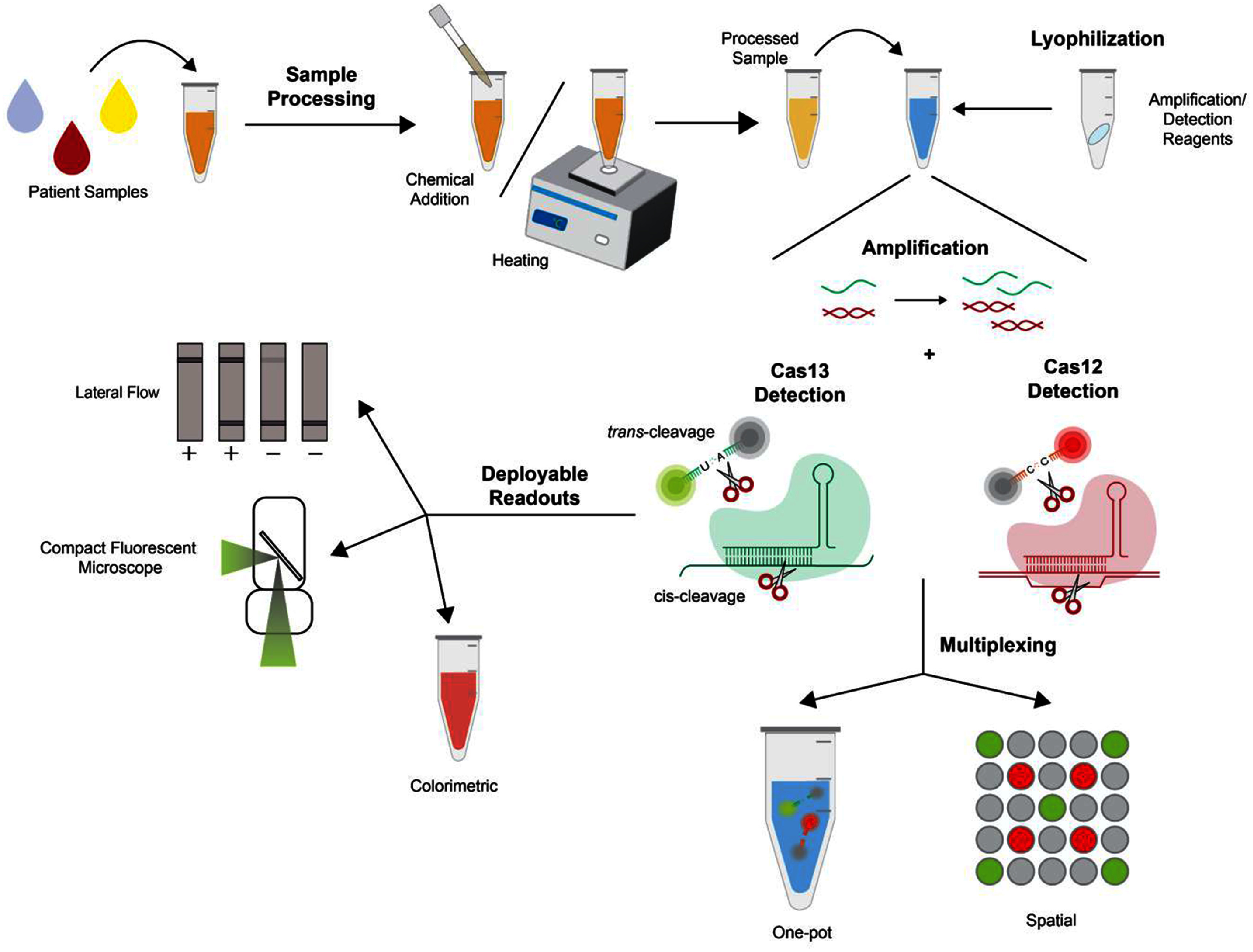
Graphical representation of subjects covered in this review. In CRISPR-Dx workflows, patient samples are first processed to inactivate pathogens and endogenous nucleases present in diverse matrices. The treated samples are then added to lyophilized CRISPR-Dx reagents, initiating amplification of the target nucleic acid sequence. Upon recognition of the amplified target, Cas enzymes activate their trans-cleavage activity, which can be coupled to specialized reporter molecules to produce detectable readouts. Multiplexing, whether performed in a one-pot format or through spatial separation, enables simultaneous detection of multiple targets. Of note, amplification can also be achieved via signal amplification, not depicted in this figure. This review highlights innovations across each of these stages in the CRISPR-Dx pipeline. See corresponding sections for further detail.

Clustered Regularly Interspaced Short Palindromic Repeats (CRISPR) technologies have the potential to address unmet needs within POC and PON diagnostics. First discovered within the adaptive immune systems of bacteria, CRISPR-Cas systems are evolutionarily diverse yet are connected by their use of CRISPR RNAs (crRNAs), which can be programmed to guide Cas ribonucleoproteins toward recognition and cleavage of DNA/RNA targets [13–15]. CRISPR-Cas systems are broadly categorized into two classes based on their Cas effector architecture: class 1 systems employ multi-protein effector complexes, whereas class 2 systems have a single effector protein [15]. Class 2 systems have emerged as key players within the field of CRISPR-based diagnostics (CRISPR-Dx). Though some CRISPR-Dx technologies use CRISPR-Cas9 [16, 17], the majority leverage CRISPR-Cas12 (Cas12), a DNA targeting system, and CRISPR-Cas13 (Cas13), an RNA targeting system. Cas12 and Cas13-based detection rely not only on crRNA complementarity for target recognition, but also the ability of Cas12 and Cas13 to collaterally cleave, or ‘*trans*-cleave’, non-target RNA molecules [18] (figure 1). Early examples of CRISPR-Dx, such as DNA Endonuclease-Targeted CRISPR Trans Reporter (DETECTR) and Specific High-Sensitivity Enzymatic Reporter UnLOCKing (SHERLOCK), leveraged the *trans*-cleavage activities of Cas12 and Cas13 by pairing them with DNA or RNA reporter molecules whose cleavage would produce detectable signal [19, 20]. Driven by the specificity of CRISPR-Cas effector activation, SHERLOCK and DETECTR are capable of distinguishing differences at the single-nucleotide level, exhibiting significantly greater sensitivity than isothermal amplification methods [19, 21]. However, signal generation consists of only one part of their workflows. DETECTR and SHERLOCK require additional steps for successful nucleic acid detection, many of which are not optimized for POC or PON use. For example, sample processing remains a key limitation for both protocols. SHERLOCK relies on laboratory-based commercial kits to lyse pathogens and extract nucleic acids prior to recombinase polymerase amplification (RPA) and Cas13 detection, significantly limiting accessibility in both POC and PON settings. In contrast, DETECTR employs a simplified heat treatment followed by proteinase K digestion to process fecal samples for human papillomavirus (HPV) detection; however, this approach is not readily adaptable across diverse sample matrices or pathogen types. Though these early diagnostics were foundational for the CRISPR-Dx field, their workflows were not adapted for field deployment.

The onset of the COVID-19 pandemic catalyzed intensive efforts to optimize CRISPR-Dx technologies, and in the years since, significant advancements have rapidly moved these technologies toward both POC and PON deployment. In this review, we highlight these advances by focusing on improvements in sample processing, isothermal and amplification-free detection methods, and multiplexing strategies (figure 1). We also discuss additional developments that enhance CRISPR-Dx POC and PON deployment, including advances in reagent lyophilization and signal readout technologies (figure 1). Lastly, we provide an overview of open challenges and opportunities for future innovation.

## 2. Sample processing

Sample processing is an important step in CRISPR-Dx workflows and affects downstream nucleic acid amplification as well as target detection. Traditional nucleic acid diagnostics, such as quantitative PCR (qPCR) or reverse-transcription qPCR (RT-qPCR), have relied on conventional nucleic acid extraction workflows for sample processing, using either solid-phase or liquid-phase techniques to achieve sample lysis, nuclease inactivation, and purification [22–25]. While effective, these methods require laboratory equipment, are time-consuming, and involve multiple handling steps. Thus, conventional sample processing methods significantly limit assay deployment. As such, streamlining sample processing remains essential for advancing CRISPR-Dx accessibility.

The ideal sample processing method for POC and PON CRISPR-Dx would enable rapid, equipment-free nucleic acid isolation while simultaneously inactivating pathogens, reaction inhibitors, and nucleases that could cause non-specific reporter cleavage. However, achieving effective inactivation is difficult in light of the unique sample processing challenges associated with diverse biological matrices. For example, in saliva and sputum samples, glycoproteins such as mucins greatly increase sample viscosity, which can hinder efficient sample lysis, sequester polymerases needed for nucleic-acid amplification, and lower overall detection kinetics [26–28]. Urine and stool samples contain high levels of protein denaturants, such as urea and bile salts, which also interfere with polymerase stability [29, 30]. In whole blood samples, hemoglobin has been shown to decrease target amplification efficiency for other nucleic acid tests and cause fluorescence quenching, affecting diagnostic readouts [31]. As such, there is an unmet need for simple sample processing methods compatible with multiple matrices.

Sample processing methods must also account for different types of pathogens. Viruses can be characterized by structurally resilient protein capsids that require strong reducing or chaotropic agents for full nucleic acid extraction [17, 32]. Gram-positive bacteria possess thick, highly cross-linked peptidoglycan walls that are commonly resistant to typical enzymatic, mechanical, or chemical treatments [33]. For parasites that undergo intracellular life stages, effective nucleic acid extraction requires lysis of both the parasite and host cell membranes [34]. Because of the unique challenges associated with different matrices and pathogens, sample processing approaches for CRISPR-Dx are typically tailored to specific applications and developed by trial and error. A method that is generalizable across the matrix-pathogen space would greatly simplify CRISPR-Dx workflows and increase their adaptability, and this remains a major outstanding challenge for the field to address.

### 2.1. Chemical-based sample processing methods

**Figure 2.**
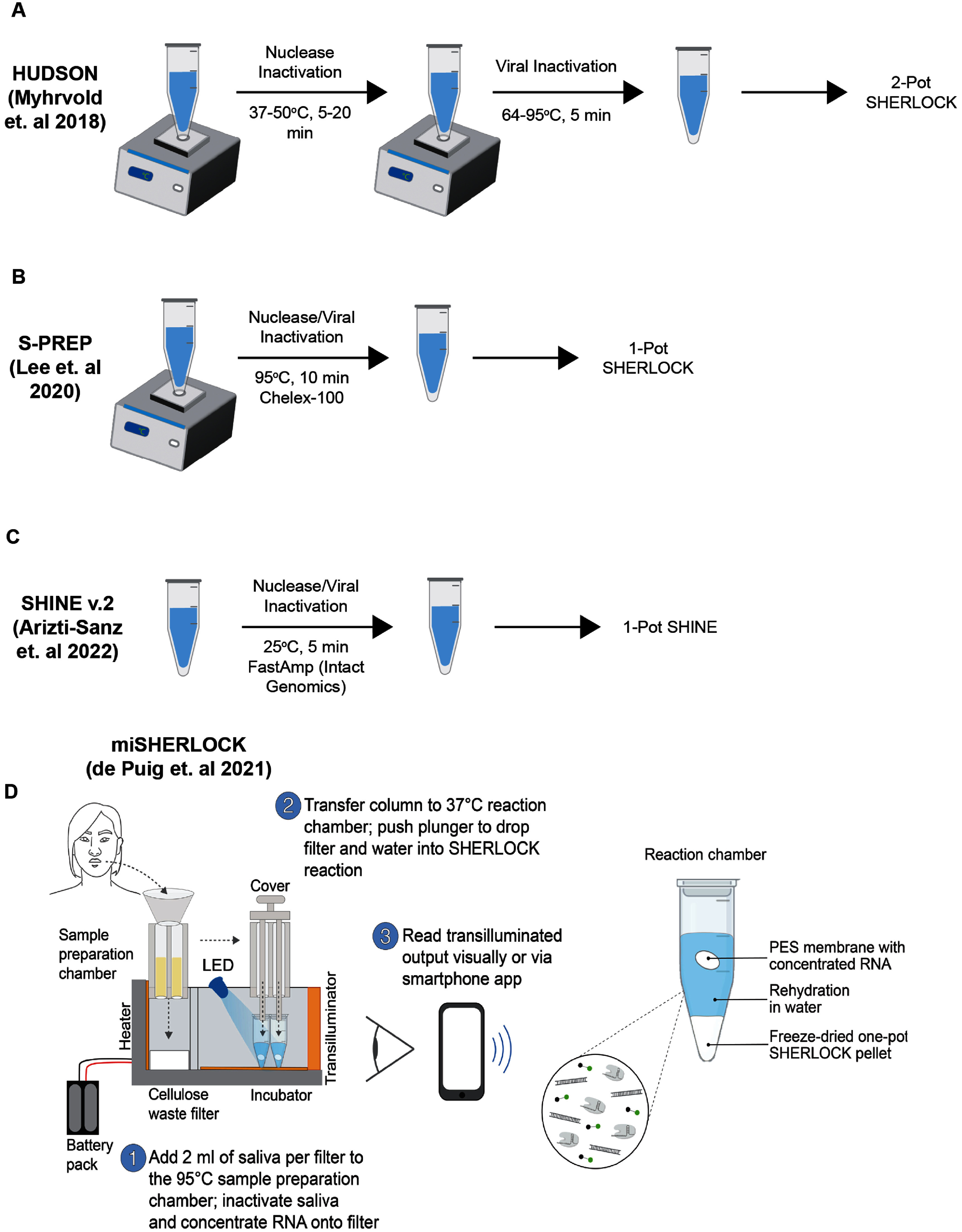
Schematic representation of sample processing methods. **A.** HUDSON protocol. Samples are subjected to a two-step heating treatment for pathogen and nuclease inactivation [35]. **B.** S-PREP protocol. Samples are subjected to a one-step heating treatment. Chelex-100 is introduced to inactivate nuclease rich environments [36]. **C.** SHINE V.2 protocol. FastAmp (intact genomics) reagents are used to lyse samples at room temperature [40]. **D.** miSHERLOCK protocol. miSHERLOCK integrates sample processing and detection into an automated workflow. From [43]. Reprinted with permission from AAAS.

In recent years, efforts have been made to develop chemical-based sample processing methods that are compatible with multiple matrices and pathogens (table 1). These approaches largely rely on protocols where the sample is incubated in a buffer that inactivates nucleases, lyses pathogens, and can be directly used as input for downstream detection. For instance, the HUDSON protocol (heating unextracted diagnostic samples to obliterate nucleases), which was initially designed for use with SHERLOCK, achieves nuclease inactivation and pathogen lysis through chemical reduction with tris(2-carboxyethyl)phosphine hydrochloride (TCEP), ion chelation with ethylenediaminetetraacetic acid (EDTA), and two heating steps: an initial incubation at 37 °C–50 °C for 5–20 min followed by a second incubation at 64 °C–95 °C for 5 min [35] (figure 2(A)). HUDSON enabled detection of both Zika Virus (ZIKV) and dengue virus (DENV) in matrices including urine, whole blood, plasma, serum, and saliva samples. However, HUDSON was ineffective for *Plasmodium* nucleic acid extraction and could not sufficiently inactivate nucleases from whole blood samples [36]. To detect *Plasmodium* species, S-PREP (SHERLOCK rapid parasite extraction protocol) was developed, in which samples are incubated at 95 °C for 10 min in a buffer containing Chelex-100 resuspended in TE with DTT (figure 2(B)). S-PREP was capable of detecting *Plasmodium* in whole blood, plasma, and urine. The addition of Chelex-100, a stronger chelating agent than EDTA, was likely responsible for successful inactivation of nucleases, which require ions as cofactors for activity, and has since been used in other sample preparation methods for difficult-to-lyse pathogens such as *Mycobacterium tuberculosis* [37, 38]. Commercial sample processing kits have also been tested for compatibility with CRISPR-Dx. The QuickExtract Kit (Lucigen) was previously shown to facilitate rapid SARS-CoV-2 nucleic acid extraction from nasopharyngeal swabs, using a single heating step at 95 °C for five minutes [39]. While the QuickExtract protocol improves on HUDSON and S-PREP by decreasing sample processing time, further validation is needed to confirm its compatibility with other matrices and pathogens.

**Table 1.** Chemical-based sample processing methods.

Sample processing method	Heating steps	Buffer composition	Additional equipment	Time	Pathogens detected	Sample compatibility	Number of patient samples tested	Reference
HUDSON	Incubation 1 (37 °C–50 °C) Incubation 2 (64 °C–95 °C)	TCEP, EDTA	Dry Heat Block	10-25 min	ZIKV, DENV	Urine, whole blood, saliva, serum, plasma	53	35
S-PREP	Incubation 1 (95 °C)	Chelex-100, TE, DTT	Dry Heat Block	10 min	Plasmodium	Urine, whole blood, plasma	15	36
STOPCovid.v2	Incubation 1 (95 °C)	QuickExtract (Lucigen)	Dry Heat Block	5 min	SARS-CoV-2	Nasopharyngeal swabs	402	39
SHINE v.2	Room Temperature (25 °C)	FastAmp Reagent (Intact Genomics)	None	5 min	SARS-CoV-2	Nasopharyngeal swabs	72	40
mutaSCAN	Room Temperature (25 °C)	Triton X-100, betaine, glycerol, Tween-20, DTT	None	1 min	SARS-CoV-2	Nasopharyngeal swabs	48	41
EXORCA	Room Temperature (25 °C)	8× Kushan Nucleic Acid Releasing Reagent (Beijing Baoying Tonghui Biotechnology)	None	1 min	Klebsiella pneumoniae	Sputum, bronchoalveolar lavage, whole blood	20	42

Protocols such as HUDSON, S-PREP, and QuickExtract represent important steps toward simplifying CRISPR-Dx workflows in POC settings, yet their reliance on heating elements prevents their full adoption in PON settings. To address these needs, streamlined highlighting of infections to navigate epidemics (SHINE) v.2 was developed, which utilizes a buffer consisting of FastAmp reagent (intact genomics) and RNase inhibitors to achieve SARS-CoV-2 lysis in five minutes at room temperature [40] (figure 2(C)). SHINE v.2 was performed on nasopharyngeal swabs and achieved 90.5% sensitivity and 100% specificity. More recently, Zhang *et al* developed mutaSCAN (microfluidic multiplate-based ultrahigh throughput analysis of SARS-CoV-2 variants using CRISPR/Cas12a and Nonextraction RT-LAMP) for room temperature extraction and detection of SARS-CoV-2 variants [41]. Unlike SHINE v.2, which relies on commercial products, mutaSCAN uses a homemade lysis buffer containing Triton X-100, betaine, glycerol, Tween-20, and DTT. The authors also found sample lysis with this buffer could be sufficiently achieved in 1 min at room temperature, suggesting enhanced performance over SHINE v.2. For detection of *Klebsiella pneumoniae*, Fu *et al* developed EXORCA (Extraction-free One-pot RPA-CRISPR/Cas12a Assay) which uses the 8X Kushan nucleic acid releasing reagent (Beijing Baoying Tonghui Biotechnology Co., Ltd) with pretreatment of sputum, bronchoalveolar lavage fluid, and blood samples for 1 min at room temperature [42]. By eliminating heating steps during sample extraction, technologies like SHINE v.2, mutaSCAN, and EXORCA help minimize equipment needs for CRISPR-Dx. Evaluating their compatibility with other samples and pathogens would further expand CRISPR-Dx in POC and PON settings.

### 2.2. Integrating sample processing and detection

While solution-based methods greatly simplify nucleic acid extraction for CRISPR-Dx, they still require the transfer of processed samples into a separate tube for subsequent detection. In non-laboratory settings, such transfers introduce contamination risk that could lead to false positives. An ideal sample-to-answer CRISPR-Dx would integrate sample processing with detection into a single reaction to minimize handling steps. However, attempts to accomplish a one-pot sample-to-answer assay at room temperature have been limited, as sample-processing reagents often interfere with downstream detection when combined. Engineering solutions offer a promising alternative to simplify the sample processing workflow. Developed during the height of the COVID-19 pandemic for SARS-CoV-2 detection, miSHERLOCK (minimally instrumented SHERLOCK) is a low-cost (<$15), easy to use POC diagnostic that combines sample processing, amplification, detection, and readout in one platform [43] (figure 2(d)). In miSHERLOCK, saliva samples, channeled through a collector, are first treated with a DTT/EGTA buffer and heated at 95 °C for six minutes to achieve viral extraction and nuclease inactivation. The released RNA is captured and concentrated onto a polyethersulfone (PES) membrane, which is transferred into a reaction chamber containing lyophilized one-pot SHERLOCK reagents. Depressing a built-in plunger punctures a water reservoir that rehydrates the lyophilized one-pot SHERLOCK reagents, initiating amplification and Cas12a-based detection. While miSHERLOCK still requires a handling step to move the PES membrane into the reaction chamber, it remains highly user friendly while avoiding any need for pipetting or laboratory-based techniques. More recently, Lee *et al* developed CreDiT (CRISPR enhanced digital testing) HPV testing [44]. CreDiT combines both nucleic acid extraction and CRISPR detection in one tube, requiring only one in-between manual step. The sample is first incubated in a lysis buffer containing proteinase K (Pro K) at 56 °C for 15 min, followed by Pro K deactivation at 95 °C for 1 min. After deactivation of Pro K, the user manually adds CreDiT reagents for RPA and Cas12-based detection directly to the sample tube, initiating the reaction for eventual fluorescence readout. These sequential heating steps and reactions are automated by a customized instrument. While one handling step is necessary for CreDiT, this can be streamlined by a new tube design where two compartments are separated by a breakable seal. Platforms such as miSHERLOCK and CreDiT are difficult to deploy in PON settings due to instrumentation requirements. However, in other POC settings such as in clinics, these engineered solutions can enable rapid testing while providing a level of accuracy often lacking in PON tests.

## 3. Isothermal amplification and amplification-free methods

Nucleic acid based tests often rely on target amplification to increase assay sensitivity and specificity. Early CRISPR-Dx workflows incorporated nucleic acid amplification as a separate step, performing amplification and target detection in distinct reactions. However, such multi-step workflows are poorly suited for POC and PON applications. Besides increasing operational complexity, multi-step CRISPR-Dx workflows also introduce contamination risk by requiring transfer of materials between amplification and detection reactions. As such, substantial effort has been directed toward developing one-pot CRISPR-Dx technologies that either integrate amplification and detection steps in a single reaction, or circumvent the need for amplification altogether. This section highlights recent advances in one-pot CRISPR-Dx technologies, examining both isothermal amplification–based platforms and amplification-free approaches, including autocatalytic systems, microconfinement strategies, and multilinear signal amplification.

### 3.1. Isothermal amplification approaches

In recent years, there have been new methods to try and improve isothermal amplification to allow for minimal liquid handling steps and increased sensitivity. This is increasingly important in one-pot formats as Cas12 is known to interfere with amplification by cleaving the target molecules before sufficient amplification has occurred [45–47]. As such, many groups have been optimizing one pot reactions using isothermal amplification. RPA is popular amongst CRISPR-Dx due to its similar working temperatures to various Cas enzymes (37 °C) [48]. This is the case for both Cas12 [46, 49–53] and Cas13 [37, 40, 47, 54, 55]. Tong *et al* improved upon one-pot testing for both AapCas12b and LwaCas13a [47]. To optimize AapCas12b one-pot reactions for detection of DNA targets, they created a mutant enzyme, and for LwaCas13a one-pot they adapted SHINE to instead recognize the amplified reverse complement of viral RNA. Cheng *et al* found that adding heparin sodium to one-pot RPA based reactions can allow for detection of suboptimal PAMs and increase sensitivity by preventing Cas12a from cleaving before sufficient amplification can occur [49]. This work achieved maximum DNA reporter cleavage and minimal amplification inhibition by including multiple RNPs with gRNAs that favor *trans*- vs *cis-*cleavage. Another method, thermally regulated asynchronous CRISPR-enhanced, segregates the amplification and detection steps in a one-pot by using temperature dependent inhibitors [50]. Other techniques rely on Cas13a, even for DNA targets, opting for its high collateral activity without degradation of the initial target prior to adequate amplification [37, 55]. Several groups have deployed photoactivated techniques to allow for timed separation between amplification and detection in a one-pot format, where sufficient amplification occurs before using light to allow detection to proceed [51–53]. These methods have attempted to overcome shortcomings associated with one-pot RPA CRISPR-Cas reactions.

Loop-mediated isothermal amplification (LAMP) also shows great promise for one-pot CRISPR-Cas diagnostics [56]. LAMP has also been combined with photocleavable substrates to control CRISPR-Cas activity [57]. LAMP has higher working temperatures than RPA (60 C), and thus is incompatible with a one-pot format for enzymes that work at lower temperatures, but several groups have employed AapCas12b for LAMP-based techniques as this enzyme functions at 60 C [39, 58, 59]. It is worth noting that several groups have also worked towards overcoming handling, multiplexing, readout limitations by creating isothermal amplification based methods paired with their own portable devices [44, 55, 58–60]. Custom fabricated devices pose their own challenges around usability, reliability, and cost, but they demonstrate a positive step towards user-friendly assays with minimal instrumentation requirements.

Other isothermal techniques, such as nucleic acid sequence-based amplification (NASBA), are also occasionally employed [61–64]. NASBA requires longer amplification times and is also subject to non-specific amplification [62]. Non-specific amplification is a shortcoming of all isothermal techniques, which makes it even more important to have an optimally designed CRISPR-Dx as a second level of detection control [12, 65–67]. Collectively, these works demonstrate steps towards POC CRISPR-Dx by making technological advancements that improve one-pot detection sensitivity.

### 3.2. Autocatalytic approaches

**Figure 3.**
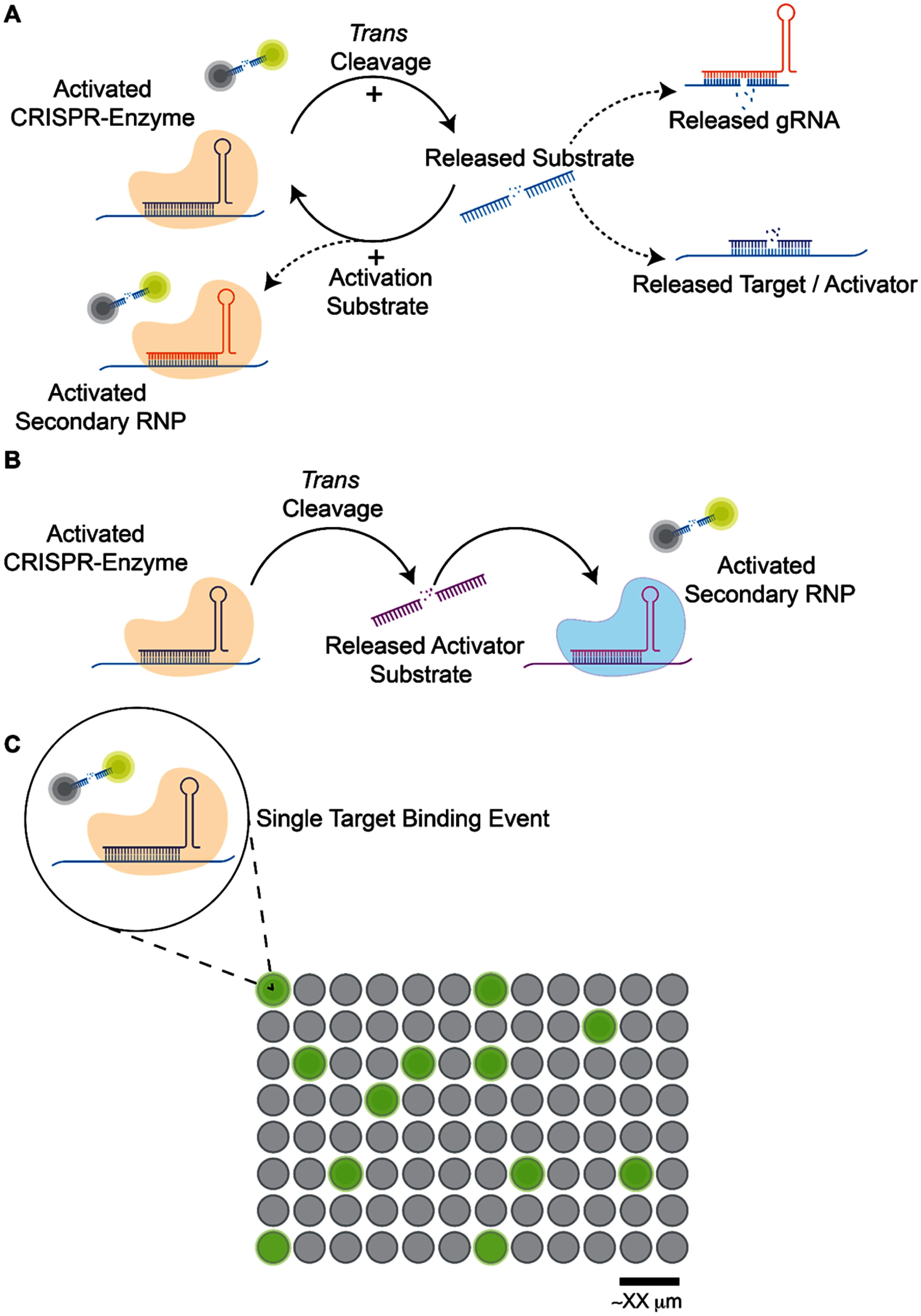
Schematic representation of nucleic acid amplification free methods. **A.** Graphical representation of CRISPR-Dx autocatalytic methods [68–73]. A positive feedback loop is created to amplify a signal. Dashed lines represent possible components of the feedback loop. **B.** Graphical representation of CRISPR-Dx multilinear methods [74–76]. **C.** Graphical representation of CRISPR-Dx microconfinement methods [77–79]. The diagrams represent some of the key strategies used, but do not encompass all methods. Released substrates may be a variety of activators and secondary RNP may be various enzymes or orthologs. Please see specific works for details [68–76].

Autocatalytic approaches circumvent the need for amplification by creating a cascade or positive feedback loop to amplify fluorescent signals. Foregoing nucleic acid amplification and instead opting for signal amplification circumvents non-specific nucleic acid amplification as well as costs associated with isothermal amplification components. Frequently, these methods leverage Cas12 and Cas13 *trans* cleavage activity to not only cleave reporter molecules but also release more, newly accessible activator substrates [68–73] (figure 3(A)). Importantly, an exponential signal amplification is established as positive feedback loops are created.

One version of DNA substrate-mediated autocatalysis of CRISPR/Cas12a (DSAC) relies upon Cas12a *trans*-cleavage to linearize a DNA substrate from its initial secondary structure, thus allowing its detection by a second crRNA to amplify signal [68]. Cas12a circular DNA amplification reaction (Auto-CAR) similarly linearizes an activator upon cleavage of a small circular DNA nanostructure (cir-mediator), and, depending on format, has a second set of ribonucleoproteins to detect this cir-mediator [69]. In one of these formats, the reporter is integrated into the cir-mediator to allow release of activator and signal simultaneously. While both assays show success in clinically relevant samples and compatibility with lateral flow, they also discuss issues with background signal and require careful consideration of signal to noise. CRISPR-Cas-only amplification network (CONAN) utilizes similar ideas to DSAC and auto-car except its positive feedback loop relies on release of a caged gRNA which targets a separate, dsDNA assistant probe [70]. As Cas12 can interfere with amplification, these autocatalytic approaches instead leverage Cas12 *trans*-cleavage of assay components to create positive feedback loops that bypass isothermal approaches that can be expensive or require extensive primer optimization.

Cas13 has also been used to create autocatalytic circuits that create more target activators [71–73]. A proof-of-concept one-tube target amplification-free Cas13a autocatalytic circuit system utilizes not only LwaCas13a but also T4 kinase, DNA polymerase, and T7 RNA polymerase along with a dsDNA probe that is composed of the reporter as well as a DNA trigger [71]. In this loop, these enzymes work together to create more RNA templates from this DNA trigger. While this assay was not tested on positive patient samples, pre-amplification-free ultra-fast and ultra-sensitive point of care testing (PASSPORT) demonstrated feasibility with both patient samples and lateral flow [72]. This assay includes the target-specific crRNA, as well as a U-rich RNA substrate and a corresponding crRNA, whereby activation of LwaCas13a by its target of interest leads to subsequent cleavage of the stem-loop structure of a U-rich RNA substrate in order to make it available for activating more LwaCas13a enzymes [72]. CRISPR anti-tag-mediated room-temperature RNA detection leverages a similar concept to PASSPORT except it relies on a single Cas13/crRNA pairing [73].

Autocatalytic approaches provide signal amplification without nucleic acid amplification which is beneficial to one-pot formats. As autocatalysis entails a small amount of target nucleic acid providing large amounts of signal, intuitively this may mean that high background or false positives may be more common as once the feedback loop is started it will cause a cascade. Unlike nucleic acid amplification reactions, which use a polymerase to produce an amplicon, autocatalytic methods use degradation as their mechanism and are thus more susceptible to being triggered in the absence of target nucleic acids as all nucleic acids will degrade spontaneously at some rate. For example, imperfections in nucleic acid synthesis or degradation of activators could cause a false start of these loops, making it ever more important to study stability and robustness of these assays over time.

### 3.3. Multilinear and linear approaches

In addition to advances in autocatalysis, multilinear and linear signal amplification is another option that avoids nucleic acid amplification and does not involve a positive feedback loop. The multilinear approach relies on sequential steps to amplify signal, rather than feedback loops [74–76] (figure 3(B)). Fast integrated nuclease detection in tandem (FIND-IT) uses both LbuCas13a and Csm6 along with a Cas13a cleavable Csm6 activator in order to detect SARS-CoV2 RNA [74]. FIND-IT employs a pool of 8 different LbuCas13 guide RNAs to achieve high sensitivity, upon initiation of *trans*-cleavage a stabilized activator will be cleaved to an appropriate length to work as a Csm6 activator, which in turn cleaves fluorescent reporter. Another SARS-CoV2 test, CRISPR LFA enabling at-home RNA testing (CLEAR), relies on LwaCas13a activation to cleave a cascade probe to release a trigger DNA that will in turn activate LbCas12a, that then cleaves a lateral flow reporter [75]. While FIND-IT and CLEAR use multilinear signal amplification by harnessing two separate CRISPR enzymes, competitive full-sized and split crRNAs have been used to achieve a similar effect [76]. Only after Cas12 has activated with its full-length crRNA/target pair is a split crRNA able to replace the full-length crRNA and complex with its split target (ssDNA target) to begin a second round of *trans*-cleavage. By including a ssDNA activator that is also complementary to the seed region of the target crRNA, they found they could directly detect RNA with Cas12 only in the presence of both the RNA and activator. While FIND-IT is compatible with a compact fluorescent detector and CLEAR is compatible with lateral flow, the asymmetric cleavage assay lacks a deployable readout [74–76]. Fozouni *et al* developed an assay that uses a linear signal amplification approach, relying on multiple crRNAs and fluorescence measurements quantified over time in order to determine viral load [80]. They demonstrated compatibility with mobile phone devices, yet the method still requires laboratory-based nucleic acid extraction and is quite sensitive to inhibitors and nucleases. Leaky activity in multilinear and linear formats may cause issues with specificity but without positive feedback loops may avoid signal runaway. These methods harness the power of multiple CRISPR enzymes in order to create a signal amplification cascade that demonstrates the benefit of multilinear approaches. Amplification free methods are more restricted in their assay formats as it is challenging to incorporate RT without nucleic acid amplification. As such, while amplification free methods avoid non-specific nucleic acid amplification associated with isothermal methods, individual methods are generally specific to either DNA or RNA.

### 3.4. Microconfinement approaches

Microconfinement can improve sensitivity by enabling digital detection, where a sample is partitioned into thousands of single target binding events that are contained (droplet or chamber) on microfluidic chips that are then screened for positive fluorescence indicating presence of the target [77–79] (figure 3(C)). CRISPR-based amplification-free digital RNA detection technologies (SATORI) used microchamber array technology to detect SARS-CoV-2 without the need for amplification but found this method to be ∼10^3^× less sensitive than amplification based methods [77]. More recently, Shinoda *et al* developed opn-SATORI to overcome this sensitivity issue by using LtrCas13a and magnet-bead based technology but this platform is large and requires both a fluorescent microscope and dispensing robot [78, 79]. While these microfluidic based approaches require a fluorescent microscope and image analysis, equipment burdens can be decreased by using a compact or portable fluorescent microscope.

### 3.5. Comparing isothermal amplification and amplification free workflows

One-pot CRISPR-Dx workflows that leverage isothermal amplification-based or amplification-free strategies have different strengths and tradeoffs. Isothermal amplification-based CRISPR-Dx are generally less susceptible to nonspecific signal generation than amplification-free platforms. However, integrating isothermal amplification with CRISPR detection presents challenges, as CRISPR enzymes can prematurely cleave amplification products before adequate amplification has occurred. Additionally, the primer optimization process for isothermal amplification can complicate assay design and optimization, and the use of additional enzymes and reagents can increase overall the costs of the assay. Autocatalytic approaches bypass the technical challenges associated with combining isothermal amplification and CRISPR detection. However, because these approaches rely on positive feedback loops for signal generation, nonspecific target cleavage can trigger runaway signal amplification, leading to false-positive interpretations and limiting assay specificity and accuracy. Multilinear or linear strategies often suffer from issues with sensitivity, especially at low target concentrations where signal generation can be difficult to distinguish from background noise, or in samples with inherently high background interference. Moreover, their use of multiple CRISPR systems increases the cost of assay development. Finally, though microconfinement approaches bypass amplification requirements, their limited sensitivity and reliance on microfluidic and imaging technologies hinder POC and PON deployment. Future efforts should focus on developing one-pot CRISPR-Dx platforms that combine the sensitivity and specificity characteristic of isothermal amplification approaches with the simplicity of amplification-free methods, while also improving overall workflow speed.

## 4. Multiplexing

The ability to detect and discriminate between multiple diagnostic targets can often be crucial for effective clinical decision-making, such as when different pathogens exhibit overlapping symptoms or variants of the same pathogen carry mutations that confer drug resistance [81–83]. In these scenarios, relying on single-target assays can delay initiation of appropriate therapies, increase frequency of unnecessary treatments, and hinder overall epidemiological surveillance [10]. By enabling simultaneous detection of multiple targets, multiplexed diagnostics address these limitations of single-target assays. As such, the development of multiplexed CRISPR-Dx has emerged as a focal point for innovation (table 2).

**Table 2.** **Multiplexed CRISPR-Dx platforms.** (1) Sensitivity reported in paper as 2 aM (≈1.2 copies *µ*l^−1^ ). (2) Sensitivity reported in paper as 1 aM (≈0.6 copies *µ*l^−1^ ). (3) Sensitivity reported in paper as 0.26 aM (≈0.15 copies *µ*l^−1^ ). *The paper does not clearly specify whether analytical sensitivity is defined with respect to the original input concentration or the effective concentration after addition to the reaction mixture.

Multiplexing technology	Multiplexing approach	Number of reactions	Analytical sensitivity	Time (min)	Reference
SHERLOCK v.2	Orthogonal cleavage	4	1.2 copies/*µ*l^1^	60	86
OPTIMA-dx	Orthogonal cleavage	2	10 copies *µ*l^−1^	60	90
SHINE Influenza	Orthogonal cleavage	2	100 copies *µ*l^−1^	90	91
MACRO	Portable microfluidic	5	0.6 copies/*µ*l^2*^	45	96
LOC-CRISPR	Portable microfluidic	10	100 copies *µ*l^−1^	60	95
MiCaR	Portable microfluidic	30	0.15 copies/*µ*l^3*^	35	94
mSHINE	Portable microfluidic	112	10 copies *µ*l^−1^	60	97

**Figure 4.**
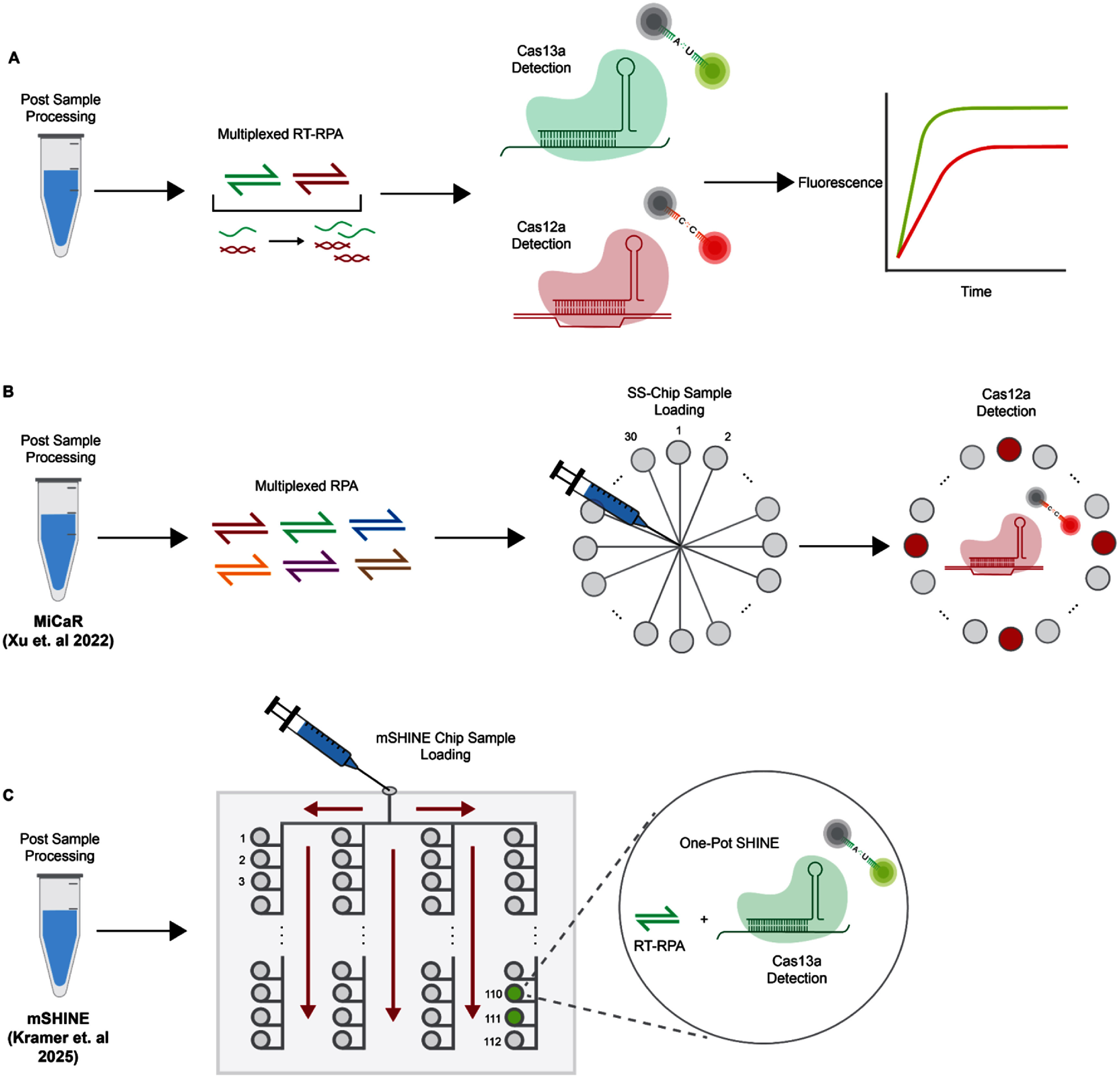
Schematic representation of multiplexed detection. **A.** Orthogonal collateral-cleavage based multiplexed detection with Cas13a and Cas12a. **B.** MiCaR workflow for HPV diagnosis. MiCaR can achieve up to 30-plex reaction partitioning, however it is also limited by the multiplexing capacity of RPA. Reproduced from [91], with permission from Springer Nature. **C.** mSHINE workflow. mSHINE can achieve up to 112-plex reaction partitioning. Reproduced with permission from [92].

Initial efforts to design multiplexed CRISPR-Dx harnessed the orthogonal collateral-cleavage base preferences of different Cas effectors to activate reporter molecules unique to each target [84, 85] (figure 4(A)). SHERLOCK v.2 was one of the initial platforms to leverage this strategy [85]. SHERLOCK v.2 achieved detection of four separate targets by combining LwaCas13a, CcaCas13b, PsmCas13b, and AsCas12a in a single reaction. In this system, Cas13 enzymes were used to collaterally cleave ssRNA reporters, whereas Cas12 collaterally cleaved ssDNA reporters. By designing reporters with fluorogenic dyes and nucleic acid linker compositions unique to each Cas effector, SHERLOCK v.2 enabled differential readout for each target in one reaction. Though SHERLOCK v.2 was used for detection of ZIKV and DENV viruses, similar approaches have been adopted for various other pathogens [86–88]. OPTIMA-dx, which was developed during the COVID-19 pandemic, combines a thermostable TccCas13a and AapCas12b for SARS-CoV-2 identification in a one-pot, single-temperature reaction [89]. TccCas13a was used to detect the virus, and AapCas12b was used to detect an RNAse P internal control, which helps indicate proper sample handling, RNA extraction, template quality, and validity of reagent. More recently, SHINE was adapted for simultaneous detection of influenza A virus by LwaCas13a and RNase P by LbaCas12a [90]. These platforms support multiplexed detection, but their capacity remains limited to only a few (two to four) targets. This constraint is explained in part by the number of Cas effectors with sufficiently different collateral cleavage preferences available for detection. Though the discovery of additional Cas enzymes could, in principle, expand this strategy’s multiplexing potential, other factors, such as the number of spectrally distinct reporters or upstream amplification of multiple targets, act as additional bottlenecks. While some diagnostic applications can benefit from this multiplexing strategy, it would be insufficient for other tasks such as variant detection where dozens of mutations, or more, may need to be profiled.

Efforts to overcome these limitations have led to spatially multiplexed CRISPR-based nucleic acid detection platforms such as CARMEN, which uses emulsified, color-coded droplets containing samples and CRISPR reagents that are pooled, arrayed on a microfabricated chip, merged electrically, and imaged by fluorescence microscopy [93]. Although capable of detecting 169 targets across eight samples, its reliance on specialized equipment and an 8–10 h workflow limits its clinical applicability, especially at the POC. mCARMEN streamlined this system by replacing emulsification and microscopy with automated mixing on a Fluidigm IFC chip [94]. mCARMEN enables detection of 24 targets across 192 samples in under five hours, but it still requires laboratory infrastructure and is unsuitable for POC use. Ideally, multiplexed CRISPR-Dx would couple the simplicity of orthogonal Cas systems with the high-throughput capabilities demonstrated by CARMEN and mCARMEN.

Portable microfluidic platforms have great potential to achieve highly multiplexed CRISPR-based detection in POC settings. An early example includes MiCaR (microfluidic device with CRISPR-Cas12a and multiplex RPA amplification), which uses a starburst-shaped microfluidic chip (SS-Chip) to achieve testing of up to 30 nucleic acid targets [91] (figure 4(B)). One sample is loaded into a center hub well on SS-Chip, which then uses spoke microchannels to homogeneously distribute it into 30 labeled wells pre-loaded with a LbCas12a detection mix. While highly multiplexed, MiCaR requires pooled off-chip RPA amplification prior to loading on SS-Chip, introducing complex liquid handling steps and potential sources of contamination. To address these limitations, other portable microfluidic technologies that combine both sample amplification and multiplexed detection in an enclosed system have been developed. LOC-CRISPR is a platform that integrates magnetic bead-based nucleic acid extraction, RPA amplification, and fluorescence readout all in one system, organizing each step into its own compartment and using syringe pumps to move materials between them [95]. LOC-CRISPR achieves detection of five targets for two samples while also minimizing the risk of contamination. MACRO is another microfluidic device that achieves multiplexed detection using a ‘spatial hourglass’ design where fluid movement is driven by flipping the chip rather than syringe pumps [96]. The device is preloaded with lyophilized RPA and LbCas12a reagents. Upon sample input, gravity-driven flow directs the sample through metering channels and into five distinct reaction chambers, each supporting an independent detection reaction. Platforms such as LOC-CRISPR and MACRO minimize workflow complexity by combining upstream sample processing and amplification with downstream detection. However, in comparison to MiCaR or CARMEN, the multiplexing capabilities of these platforms remains limited. Recently, SONATA (Scalable On-site Nucleic Acid Testing Architecture) was developed, which greatly increases multiplexing while also maintaining workflow simplicity [92] (figure 4(C)). SONATA utilizes a microfluidic chip of comb geometry to direct a single sample, which is injected via syringe, into more than 100 different chambers homogeneously. Each chamber contains its own pre-loaded lyophilized SHINE reagents, including RPA pellets for amplification and LwaCas13a detection assays. Upon sample entrance into each chamber, the lyophilized reagents are rehydrated and mixed via a magnetic stirring bar, enabling detection of >100 targets in less than 1 h. mSHINE currently represents the highest multiplexing capacity achieved by a portable microfluidic platform. Collectively, these advances demonstrate the potential for portable microfluidic systems to bring highly multiplexed CRISPR-Dx out of laboratories and into POC settings, enabling rapid yet comprehensive syndromic testing.

## 5. Lyophilization

Liquid reagents for CRISPR-Dx that require cold chain storage represent a significant barrier for deployment in resource limited POC settings [97, 98]. Lyophilization (freeze drying) offers a practical solution and is frequently used to stabilize reagents for long-term storage and transport. During lyophilization, a solution is frozen and subsequently exposed to a vacuum such that water and other solvents are extracted via sublimation [99]. Beyond eliminating cold-chain storage needs, incorporating lyophilization into CRISPR-Dx workflows extends reagent shelf life and streamlines use by enabling simple reconstitution of a full reaction [100]. However, lyophilization exposes reagents to destabilizing conditions. As the sample freezes, ice formation forces salts and other solutes into small pockets of unfrozen liquid, creating large local pH shifts that can trigger protein aggregation and denaturation [101]. Furthermore, removal of the hydration shell during the subsequent dehydration step further destabilizes protein and nucleic acid structures, promoting their misfolding [102].

To address these challenges, advances have largely been focused on identifying chemical additives to preserve CRISPR-Dx reagent stability during lyophilization. In S-PREP, reagents for SHERLOCK were snap-frozen in liquid nitrogen for 1 min and then lyophilized for 6 h using a Labconco freeze-dryer [36]. The authors reported that lyophilization of reagents in S-PREP led to an increase in assay sensitivity, though this was largely due to the increase in input sample volume used to reconstitute lyophilized reagents. Additionally, the shelf-life of the lyophilized reagents was not assessed, a key metric when evaluating the efficiency of a lyophilization protocol. For SHINE v.2, lyophilization of SHINE reagents directly led to an over 75% reduction in fluorescence signal post-lyophilization [40]. To improve lyophilization, PEG-8000, which can prevent efficient sublimation by retaining water, and KCl, which can alter environmental pH during freezing, was removed from the list of lyophilized products. Additionally, the authors added sucrose and mannitol as lyoprotectants, which help stabilize proteins and nucleic acids through hydrogen bonding networks and formation of protective crystalline matrices [102, 103]. These changes led to an almost complete recovery in fluorescence activity from the detection reaction. The optimized lyophilized pellets were then evaluated for shelf-life across different storage temperatures. They retained full activity for at least one month when stored at 4 °C or—20 °C. In contrast, their stability at room temperature was limited to only 1 week.

Other attempts to optimize lyophilization for increased shelf life at room temperature have yielded similar results. Shen *et al* recently developed Enhanced One-pot, helicase-assisted RPA-combined, Dual-CRISPR/uAsCas12a (EOD-CRISPR) for multiplexed bacterial detection, using a two-part lyophilization strategy [104]. The helicase-assisted RPA mixture was freeze-dried separately with trehalose and glycine, which function similarly to sucrose and mannitol. The CRISPR mixture was lyophilized in parallel with trehalose, glycine, and pullulan, with pullulan included to prevent protein aggregation via steric hindrance effects [105]. For use, the two pellets were combined with sample input to generate a one-pot assay. At 100 copies/*µ*L, EOD-CRISPR remained functional after one month of storage at -20 °C or 4 °C. However, at room temperature, assay performance declined rapidly, with reliable detection maintained for only up to 1 week. In two-pot systems, where amplification and detection are performed separately, individually lyophilizing RPA and CRISPR components with chemical additives can preserve assay performance for up to a month at room temperature [106–108]. However, two-pot systems often require multiple handling steps, complicating workflows and introducing contamination risk. Thus, there is an urgent need for methods that extend the room temperature shelf life of one-pot lyophilized CRISPR-Dx reagents.

## 6. Readout

CRISPR-Dx often employ fluorimetric readouts for signal detection. These diagnostics typically leverage reporters that carry both a fluorophore and quencher linked by ssDNA or ssRNA. Collateral cleavage of these linkers by activated Cas enzymes causes separation of the fluorophore from the quencher, generating fluorescent signals that can be interpreted by conventional plate readers, low-cost portable devices, or by the naked eye upon illumination under blue light [109–111]. While adaptable in POC settings, fluorescence-based CRISPR-Dx have limited ability for deployment in PON environments due to their reliance on additional imaging equipment. As a result, other methods have been developed for direct colorimetric visualization of CRISPR-Dx reactions. Yuan *et al* developed a platform that uses gold nanoparticles (AuNPs) connected by ssDNA or ssRNA linkers which, in the presence of target in the sample, becomes collaterally cleaved [112]. Collateral cleavage leads to separation of the AuNPs, producing a visible red color. However, in the absence of target, the AuNPs aggregate to sequester color production. Unfortunately, since low-speed centrifugation is necessary for efficient aggregation, the technology has limited deployability in PON environments. Hu *et al* developed magnetic pull-down-assisted colorimetric diagnosis based on the CRISPR/Cas12a system (M-CDC), which uses DNA-AuNP probes complementary to a biotinylated ssDNA substrate [113]. Without target DNA, the intact biotinylated linker hybridizes to the AuNP probes and binds streptavidin-coated magnetic beads in the detection tube. Subsequent application of a magnet removes the AuNPs from solution and leaves the supernatant clear. When the target is present, Cas12a-mediated trans-cleavage degrades the linker, preventing magnetic capture so that the AuNPs remain dispersed and the solution appears red. Because M-CDC requires only a simple magnet rather than centrifugation, it has potential for both POC and PON applications, though storage of AuNPs may present a challenge due to aggregation. More recently, Kim *et al* introduced TADICA, an equipment-free colorimetric CRISPR assay [114]. This system relies on a double-stranded thymine–adenine (TA) repeat sequence paired together with the dye DISC2(5), which binds to the intact TA sequences. When Cas12a is activated in the presence of a target, it collaterally cleaves the TA sequences, preventing dye binding and causing the solution to appear purple rather than the blue color observed in the absence of target. However, this approach may be susceptible to either false-positive or false-negative interpretations, as distinguishing purple and blue color changes can be challenging.

Lateral flow assays offer a compelling alternative for visualizing the collateral cleavage activity inherent in many CRISPR-Dx. Beyond eliminating the need for specialized equipment, these assays provide a simple binary readout and are generally more user-friendly. To this end, commercially available lateral flow assay systems (Millenia 1 T) have been widely adopted [85, 115–117]. These systems use reporter molecules labeled with both biotin and fluorescein amidite (FAM), along with lateral flow strips composed of three key regions: an anti-fluorescein isothiocyanate (FITC) antibody zone conjugated to AuNPs, a streptavidin capture control line, and an anti-FITC capture test line. Upon sample loading onto the lateral flow strip, the reporters bind to the FITC antibodies and migrate along the strip. When they reach the streptavidin line, intact reporters are captured via their biotin tag, forming a visible control band. In the presence of target, however, CRISPR collateral cleavage separates the biotin from the FAM–FITC antibody complex, allowing the latter to continue migrating until it is captured at the second anti-FITC line, producing a positive test band that indicates target detection.

Commercial lateral flow assays do not require equipment and are intuitive to read, yet their results are susceptible to false positive interpretations, especially at lower target concentrations where the line intensity of the test band can exhibit great variability. Several approaches have been explored to address these limitations. Yuan *et al* developed a CRISPR–Cas12a-mediated hue-recognition lateral flow assay for *Salmonella* detection that uses color changes rather than line intensity for easier interpretation of results [118]. Additionally, the color of the testing line varies with input target levels, shifting from red to orange to green and enabling a semi-quantitative readout. Other studies have used machine learning (ML) methods to minimize false positive interpretations of commercial lateral flow assays [119–121]. Xue *et al* recently developed ML algorithms for analyzing smartphone pictures of Cas13-based LFA results [122]. ML models were trained on 3146 images of LFA results, leading to classification accuracies of 96% from the ground truth. Although Xue *et al* does not compare ML performance directly against human readers, it demonstrates that ML-based interpretation can overcome common visual interpretation challenges and may enable more reliable at-home use of CRISPR diagnostic lateral flow assays. However, other limitations exist for lateral flow assays beyond false-positive interpretation. Most CRISPR-Dx that employ lateral flow readouts require additional handling steps to transfer amplification and detection products onto the lateral flow assay, introducing contamination risk. Additionally, lateral flow assay lines can change in intensity over time, meaning users must read the test at a precise time to avoid false-positive or negative readouts, which is challenging for untrained users to do.

When considering CRISPR-Dx readout methods, a current tradeoff exists between assay specificity and deployability. Fluorimetric readouts offer high accuracy and can be adapted for quantitative measurements, but their reliance on specialized imaging equipment limits their adaptability in PON settings. Similarly, many colorimetric approaches face constraints related to instrumentation requirements and reagent storage, although continued innovation may enable more deployable formats while minimizing false-positive and false-negative interpretations. In contrast, lateral flow–based CRISPR-Dx platforms are highly portable and well-suited for PON use, yet they typically provide qualitative results and can be vulnerable to interpretive variability due to differences in band intensity and user-defined thresholds. Addressing these limitations will be critical to achieving both robust performance and broad accessibility in future CRISPR-Dx technologies.

## 7. Discussion

Since the COVID-19 pandemic, there has been an increased need for deployable and reliable diagnostics. Validating and creating such diagnostics requires careful consideration of all steps along the diagnostic workflow, including sample processing, amplification, multiplexing, and deployability. This review discusses advances in each of these stages.

As the first step in the workflow, user-friendly sample processing should achieve efficient pathogen lysis and inactivation while minimizing liquid handling steps and advanced equipment. PON tests that use minimally invasive sample types, such as urine, stool, or saliva, would require quick and intuitive sample preparation that could be done at home without a heating step. This may be possible for some respiratory pathogens, such as SARS-CoV-2, that can be easily inactivated [40]. For tests that rely on more invasive sample types collected at the point of care, such as blood or sputum, more involved sample preparation, such as additional heating or centrifugation, may be acceptable [35, 36, 38, 39]. Sample processing protocols should also work for multipathogen nucleic acid extraction, given that, in real-world scenarios, a patient’s specific disease may not initially be known when processing a sample. Overall, quick, robust sample processing shows promise if paired with a one-pot, easy readout diagnostic that does not require highly purified nucleic acids.

More work needs to be done to improve the efficiency and simplicity of both one-pot format CRISPR-Dx and readout methods. While one-pot CRISPR-Dx are becoming increasingly promising, these tests still do not work as effectively as two-pot CRISPR-Dx [37]. This bottleneck, most likely attributed to *trans*-cleavage interference or mismatched ideal conditions for amplification and detection [45–47], requires continued refinement to ensure efficient amplification and high assay sensitivity. For PON diagnostics, readouts should be easily interpretable. As such, commercial-based lateral flow assays are commonly adapted for CRISPR-Dx applications [85, 115–117]. However, end-user interpretation of lateral flow assays is complicated by varying line intensities caused by variations in target concentrations. Thus, utilizing a smartphone [118], ML [122], or a color intensity reference card [123] would aid in user interpretation and improve reader agreement.CRISPR-Dx that employ colorimetric readouts are a promising alternative, though there remains a need for straightforward, equipment-free colorimetric assays. Given the development of colorimetric RT-LAMP assays [124] and recent demonstrations of CRISPR-Dx compatibility with LAMP [39, 58, 59], a straightforward colorimetric CRISPR-Dx can potentially be developed. Other options include an all encompassing stand alone device, which includes amplification and readout in an all-in-one device. Ideally such devices would also integrate sample processing. Future work to develop a simple POC or PON diagnostic should focus on easy, one-step sample preparation, one-pot amplification detection, and an integrated readout.

**Figure 5.**
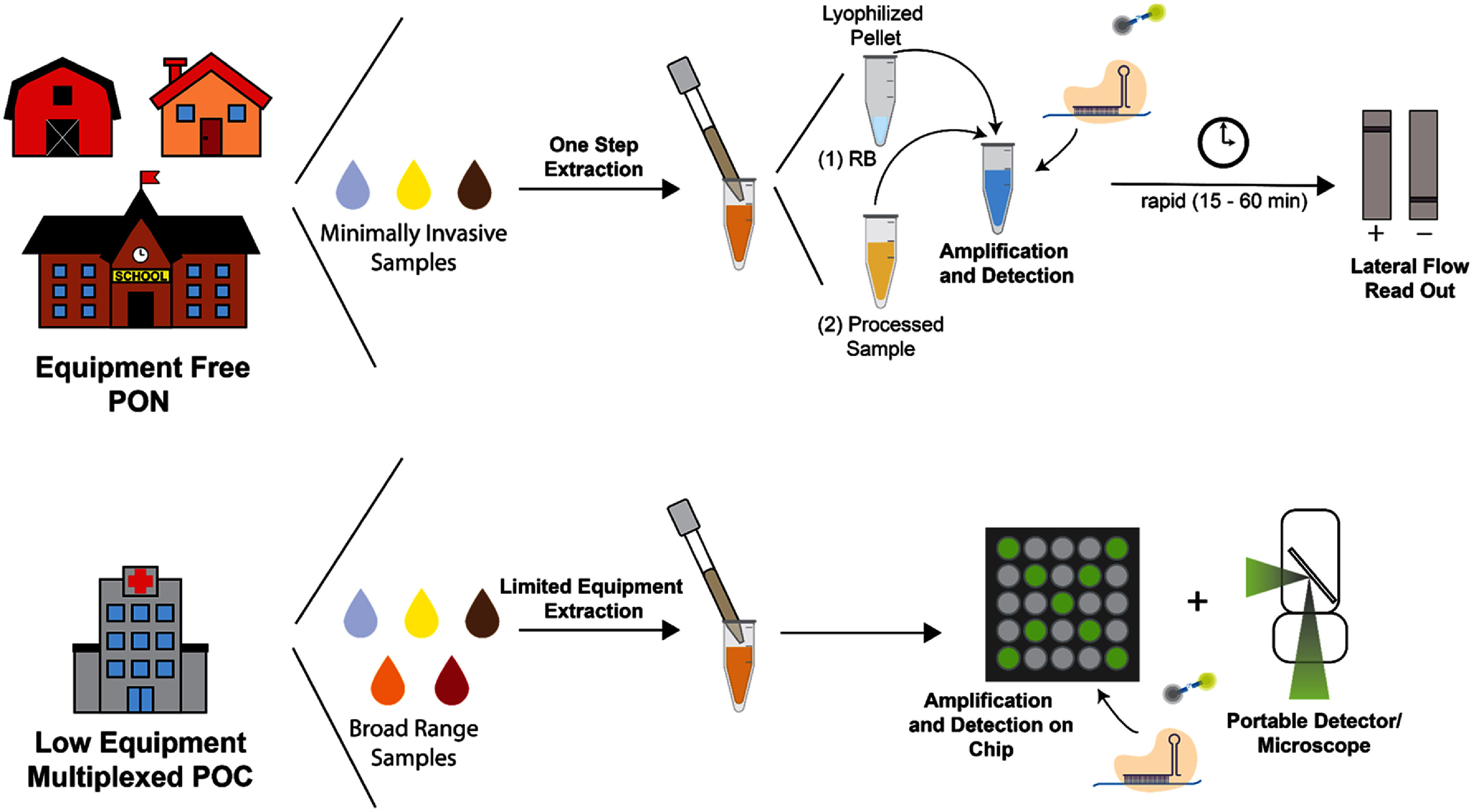
Schematic demonstrating ideal PON and POC CRISPR-Dx. Equipment free PON would be performed on-site, such as at a farm, at home, or at school. Sample types must be minimally invasive such as saliva, urine, or stool. An ideal workflow would include no pipetting, easy transfer of sample to assay, fast and easily interpreted visual readout such as lateral flow. Rapid time scale from ASSURED criteria [10]. For PON tests that also allow for more invasive sample types such as sputum or blood, a more complicated workflow may be acceptable. Low equipment multiplexed PON may require more equipment, such as a compact microscope or smartphone reader, but should still be portable and easy to use by minimally trained personnel. Amplification may describe signal or nucleic acid amplification. Extraction may be performed on the device. RB: rehydration buffer.

In settings where pathogens exhibit overlapping symptoms, such as with respiratory viruses, the ideal POC and PON test shifts from single-plex to multiplex detection. Adapting CRISPR-Dx to highly multiplexed formats provides end users with the ability to discriminate between diagnostic targets in a single assay. However, multiplexed assays often increase the amount of handling or time required for operation. As such, multiplexed assays should be developed with minimal equipment and training needed even at the POC. In the future, it will be important for groups to consider the benefits of multiplexing for their specific pathogen(s) of interest (figure 5). An ideal POC platform would have high multiplexing abilities and perform both amplification and detection on chip [92], using a colorimetric readout or a portable fluorescent microscope. Multiplexed assays provide increased information at the POC that will decrease time and cost compared to individual assays if the device is streamlined, portable, and all steps are well integrated.

Lastly, creating assays that can be deployed in real-world settings requires consideration of all previous steps and of cold-chain demands. Lyophilization is a commonly adopted method for avoiding expensive transportation and increasing assay shelf life. Ideally, assays would undergo stability testing to confirm a shelf life of several months or longer. It is important to note that there are many companies that specialize in lyophilization. Future CRISPR-Dx work could consider outsourcing such protocols to allow for optimal freeze-drying and long shelf life.

## 8. Conclusion

To-date, no CRISPR-Dx assays are FDA approved, though several laboratory-based assays received Emergency Use Authorization (EUA) during the COVID-19 pandemic^5^5U.S. Food and Drug Administration (FDA) 2022 Emergency Use Authorization (EUA) reissued letter of authorization: Sherlock CRISPR SARS-CoV-2 Kit; U.S. Food and Drug Administration (FDA) 2022 Emergency Use Authorization (EUA) reissued letter of authorization: Mammoth Biosciences DETECTR BOOST SARS-CoV-2 Kit.. One of the major challenges limiting progress in CRISPR-Dx development is the lack of cohesive, end-to-end workflows. While efforts to improve deployability have produced faster sample preparation methods, more efficient one-pot assays, and integrated devices, these components are frequently optimized in isolation rather than as part of an entire system. Consequently, future work should prioritize the development of unified, well-coordinated pipelines for pathogen detection that span sample processing through readout. For academic groups, these efforts are often complicated by the challenges of evaluating end-user satisfaction and long-term stability throughout distribution. For diagnostics that will work for real-world use, the world health organization defined criteria necessary for POC applications through ASSURED (affordable, sensitive, specific, user-friendly, rapid, equipment-free, delivered) [10, 125] [50]. For PON applications, some CRISPR-Dx methods have created devices that encompass all stages on a single platform [43, 44], but more can be done to streamline the workflows and evaluate end-user satisfaction. A proper deployable diagnostic will also be cost-efficient. Often, cost-analysis may consider only the cost of reagents or device itself, and not necessary costs related with deployment including training personnel, as these are hard to measure [10]. Therefore, determining an assay’s ease of use requires accounting for real-world deployment constraints and the time needed for training. Ultimately, continued progress towards streamlined and deployable workflows has great potential to position CRISPR-Dx technologies as paradigm-shifting tools for POC and PON diagnostics.

## Data Availability

No new data were created or analysed in this study.
